# Health related quality of life of Canary Island citizens

**DOI:** 10.1186/1471-2458-10-675

**Published:** 2010-11-05

**Authors:** Juan Oliva-Moreno, Julio Lopez-Bastida, Melany Worbes-Cerezo, Pedro Serrano-Aguilar

**Affiliations:** 1 University Castilla La Mancha, Cobertizo de San Pedro Mártir s/n. 45071; Toledo, Spain; 2 CIBER de Epidemiología y Salud Publica (CIBERESP), Parc de Recerca Biomèdica de Barcelona, Doctor Aiguader, 88; 08003 Barcelona, Spain; 3 Evaluation Unit. Canary Island Health Service, C/Pérez de Rozas, 5, 4th Floor 38004; Santa Cruz de Tenerife, Spain; 4 University Hospital Nuestra Sra. de la Candelaria, Canary Island Health Service, Carretera del Rosario 145, 38009 Santa Cruz De Tenerife, Spain

## Abstract

**Background:**

The aim of the study was to describe the health-related quality of life of Canarian population using information from the Canary Island Health Survey and three observational studies developed in the Canary Islands.

**Methods:**

A descriptive analysis was carried out on a sample of 5.549 Canarian citizens using information from 2004 Canary Island Health Survey and three observational studies on Alzheimer's disease, Stroke and HIV. EQ-5 D was the generic tool used for revealing quality of life of people surveyed. Besides the rate of people reporting moderate or severe decrease in quality of life, TTO-index scores and visual analogue scale were used for assessing health related quality of life of people that suffer a specific diseases and general population.

**Results:**

Self-perceived health status of citizens that suffer chronic diseases of high prevalence, identifies by the Canary Island Health Survey and other diseases such Alzheimer's disease, Stroke and HIV, independently examined in observational studies, are worse than self-perceived health of general population. Depression/anxiety and pain/discomfort were identified as the dimensions of the EQ-5 D with highest prevalence of problems. Alzheimer's disease and stroke were the illnesses with greater loss of quality of life.

**Conclusions:**

Health related quality of life should be integrated into a set of information along with expectancy of life, incidence and prevalence of chronic diseases for developing health policy and planning health care activities The combination of information on health related quality of life from population health surveys with data from observational studies enlarges the sources of relevant information for setting health priorities and assessing the impact of health policies.

## Background

Health is one of the main determinants of the welfare of societies. Developed countries allocate a great amount of monetary and non-monetary resources to the care of their population health. Therefore, the measurement and the analysis of the evolution of the health of a population are relevant elements for health decision-makers and for the society at large.

Traditionally, the health of a population has been measured using epidemiological indicators, morbidity (incidence and prevalence) and mortality [1]. Under the traditional biomedical model of the disease, the mortality rate and the life expectancy at birth or life expectancy at a given age have traditionally been used, together with the infant mortality rate, as the main indicators of populations' health. Although the concept of quality of life arose in the social science literature in 1920 [2], the World Health Organization's (WHO) 1947 definition of health as "a state of complete physical, mental and social welfare and not merely the absence of disease or infirmity" [3] encourages a new "psycho-social" model in which consideration is not only given to the "amount of life" but also to the preferences and perception on individuals about their own health, that is, their quality of life [4,5].

Health related quality of life (HRQOL) is a multi-attribute concept encompassing physical, mental, and social dimensions. In last decades, quality of life has increased in importance as a key health indicator for several reasons [1]. First of all, it has become increasingly clear that mortality reduction cannot be the only objective for health care systems facing mostly chronic and degenerative diseases. Secondly, it has also become clear that it is the patient, not the physician, who has the authority to judge his/her health status. Thirdly, the evolution of the economic evaluation methods of health care technologies has allowed and stimulated an increase in the interest in subjective health and quality of life of patients. As Sullivan notes [1], "Medicine's epidemiological transition from acute to chronic disease is thus prompting an epistemological transition from primarily objective to primarily subjective evidence of health and health care effectiveness. Now some of the most important patient outcomes, like patient choices before them, are valid because they are subjective". Additionally, several studies reveals that a worse HRQOL is associated with higher mortality [6-9] and a greater use of healthcare services [8,10,11].

Traditionally, populations' health surveys include questions on self-perceived health status, but recently generic instruments, such as the EQ-5 D, are increasingly included in these surveys for measuring HRQOL, . EQ-5 D has been used in specific groups and in the general population in several European countries, Japan, and United States of America [12-17] and is commonly used for describing the most commonly reported health states, for establishing the health status in the community, so that different population subgroups can be compared, for studying the association between HRQOL and age, sex, socio-economic status and disease groups, for analyzing efficacy in randomized clinical trials and efficiency in evaluations of health care technologies, and for examining the association between HRQOL and mortality risk. Additionally, there is a substantial amount of literature on descriptive studies on the HRQOL in the general population. However, very few studies have specifically reported HRQOL from representative samples of the general population jointly with HRQOL data from epidemiological studies focused on diseases of lower prevalence that are not usually identified by general health surveys.

The aim of this study was to describe the health-related quality of life (HRQOL) of Canary Islands citizens in the first years of XXI century. For this purpose, we have combined information from the Canary Island Health Survey jointly with information from observational studies.

## Methods

Our primary sources of data were the *Canary Island Health Survey *(CIHS) and three observational studies on HIV/AIDS, Alzheimer's disease and Stroke. The reason that led us to combine these sources was to show a map of the health status of the Canary Island population that would be impossible to collect only with the information contained in the Canary Island health survey since diseases with devastating effects on human health but with low prevalence at the population level, like HIV/AIDS, Alzheimer's disease and Stroke, are not captured adequately by general health surveys, e.g. differences between stages of the disease.

The CIHS was carried out in the year 2004, with a sample of 4,320 adult people residing in Canary Islands (an insular southwest region of Spain with more than 2 millions inhabitants in 2008, the 4.5 per cent of the total population of Spain). The survey included questions on self-perceived health status, chronic morbidity, habits (including feeding, physical exercise and tobacco and alcohol consumption) and socio-demographics variables (as age, gender, educational level, occupation status). We focused our interest in the most prevalent health problems according CIHS: Diabetes mellitus, rheumatism-arthritis and degenerative osteoarthritis, back pain, heart problems, osteoporosis, anxiety/depression, respiratory and digestive diseases.

The three observational studies included were carried out on behalf of the Canary Islands Health Service. The study on **Alzheimer's disease **(AD) was a cross-sectional observational study with a sample of 237 patients with AD. The interviewees lived in the Canary Islands and the patients were not institutionalised. The information was obtained via telephone interview on the main carer. The questionnaire was performed using a base questionnaire of the "Trans-national analysis of the socio-economic impact of AD in the European Union" Project. The "Clinical Dementia Rating" (CDR) was used for controlling the severity of the disease. This clinical score classify the severity of the disease into three levels: mild-moderate and severe. Fieldwork was carried out in 2001 [18]. The study was approved by the Ethics Research Committee of University Hospital Nuestra Sra. de la Candelaria.

The observational study on **HIV/AIDS **was performed as a multi-centre study in the Canary Islands using a sample of 569 patients recruited at outpatient visits. The study was approved by the Ethics Research Committee of University Hospital Nuestra Sra. de la Candelaria. Demographic and clinical data were obtained from four hospitals offering HIV outpatient services in the Canary Islands. Potential participants were randomly selected from clinical records. Patients at least 18 years old were interviewed following outpatient visits at the hospitals' centres for infectious diseases. Fieldwork was carried out between January and December, 2003 [19]. The selected criteria to create the groups in the HIV research were proposed by the Center of Disease Control and Prevention (CDC). CDC distinguishes between the following phases of disease: asymptomatic HIV, symptomatic HIV and AIDS. Unfortunately, it was not possible to distinguish between different levels of severity for diagnosed diseases in the Canary Island Health Survey.

The observational study on **Stroke **survivors was a cross-sectional study with a sample of 423 people diagnosed with stroke receiving outpatient care. Patients were recruited from five hospitals in the Canary Islands, Spain, according the year the suffered the stroke and were divided into three categories: first, second and three years survivors. The fieldwork was carried out between January and December 2004. Demographic and clinical data were collected for patients previously diagnosed with stroke or their caregivers as proxies [20]. The study was approved by the Ethics Research Committee of University Hospital Nuestra Sra. de la Candelaria.

Health Related Quality Of Life (HRQOL) was measured in CIHS and the three observational studies through a generic measure, the EQ-5 D questionnaire [21.22]. The EQ-5 D has five questions asking for a self-perceived status of five different functional conditions related to mobility, personal care, daily activities, pain/discomfort and anxiety/depression. In each dimension, the interviewed person can choose between three possible answers: 'absence of problems', 'moderate problems' and 'incapacity to perform the activity or severe problems'. A respondent health status is defined by combining one level from each of the 5 dimensions (EQ-5D). A total of 243 possible health statuses can be defined in this way. HRQOL (EQ-5D) of AD patients were assessed by the patients' caregivers, as well as in the case of stroke patients with affected level of consciousness.

In order to translate this number to a single *health score*, a 'preferences index score or tariff' is needed. Actually, there are two alternative index scores or tariffs validated in Spain, the first one based on a visual analogue scale (the VAS index score or tariff) and the second one based on the time trade-off (TTO index score or tariff [23]). The results derived from both index scores or tariffs are not directly comparable in spite of some attempts to connect them [24]. The TTO scale is frequently used, and considered a suitable alternative in the literature [25,26] because preferences are usually observed through choices between alternatives health states. The results are displayed using TTO index tariffs and the observed values in the VAS thermometer.

We performed a statistical descriptive analysis. Apart from age and sex, there were no common variables in the four databases used. Due to this fact, a multivariate analysis was unfeasible. Therefore, the study described the situation of people with diagnosed diseases, but we could not analyse the associations between those illness and other health factors like education, income status, social class, habits, etc.

## Results

Tables 1 and 2 show the presence of moderate-severe restrictions in different Health Related Quality of Life dimensions associated to the identified diseases, compared with the general population. Table 3 contains TTO index scores or tariffs results for people that suffer specific diseases and table 4 shows the corresponding index scores or tariffs for general population. Finally, Tables 5 and 6 display the results obtained through the visual analogue scale-thermometer for people that suffer a specific diseases and for the general population.

**Table 1:** Percentage of people that suffer a specific disease reporting moderate or severe problems in different Health Related Quality of Life dimensions

	Average Age(sd)	Mobility	Self-care	Usual activities	Pain/ Discomfort	Depression/Anxiety
**HIV+^i^**	**40.4(8.1)**	**18.32%**	**4.60%**	**27.94%**	**44.75%**	**51.74%**

HIV-asymptomatic	39.5 (7.8)	15.50%	4.69%	23.44%	42.19%	49.41%

HIV-symptomatic	40.6 (8.1)	14.49%	5.07%	28.26%	41.61%	46.76%

AIDS	41.8 (8.7)	26.67%	4.03%	35.33%	52.00%	60.26%

**Alzheimer disease^ii^**	**75.5 (8.5)**	**68.86%**	**84.49%**	**95.10%**	**68.57%**	**73.47%**

AD mild	73.7 (7.1)	38.78%	63.27%	93.88%	65.31%	81.63%

AD medium	75.4 (8.6)	67.03%	90.11%	95.60%	68.13%	73.63%

AD severe	76.6 (9.3)	87.63%	96.91%	98.97%	71.13%	69.07%

**Stroke^iii^**	**66.9 (12.2)**	**63.01%**	**48.39%**	**64.24%**	**71.00%**	**65.90%**

Stroke survivor first year	67.2 (11.6)	56.99%	46.24%	64.89%	68.13%	64.13%

Stroke survivor second year	67.1 (12.5)	63.00%	49.49%	64.88%	72.64%	66.33%

Stroke survivor three or more years	66.4 (12.1)	66.90%	48.28%	6.45%	70.55%	66.43%

**Diabetes Mellitus^iv^**	**63.8 (13.8)**	**38.02%**	**15.70%**	**35.64%**	**62.98%**	**43.06%**

**Rheumatism; arthritis; degenerative osteoarthritis^iv^**	**62.4 (14.9)**	**41.37%**	**14.26%**	**33.24%**	**71.65%**	**46.25%**

**Back pain^iv^**	**53.8 (17.3)**	**28.66%**	**10.49%**	**26.17%**	**61.22%**	**43.11%**

**Heart problems^iv^**	**66.1 (16.3)**	**43.53%**	**20.65%**	**38.94%**	**62.83%**	**46.45%**

**Osteoporosis^iv^**	**66.3 (12.2)**	**46.31%**	**19.70%**	**37.44%**	**75.62%**	**55.28%**

**Anxiety/depression^4v^**	**53.2 (17.3)**	**29.52%**	**10.62%**	**28.39%**	**64.56%**	**70.98%**

**Respiratory Tract Diseases^iv^**	**54.8 (19.7)**	**33.18%**	**9.81%**	**31.31%**	**58.21%**	**38.86%**

**Digestive diseases^iv^**	**52.0 (17.7)**	**27.87%**	**8.40%**	**23.62%**	**58.93%**	**43.69%**

Sources: ^i ^Observational study on HIV/AIDS (19); ^ii ^Observational study on Alzheimer's disease (18); ^iii ^Observational study on Stroke (20); ^iv ^Canary Island Health Survey

**Table 2:** Canary Island General population-Percentage of people reporting moderate or severe problems in different Health Related Quality of Life dimensions

Population	Mobility	Self-care	Usual activities	Pain/Discomfort	Depression/Anxiety
General population	16.18%	5.63%	13.19%	36.38%	27.03%

General population (men)	13.16%	4.73%	10.70%	27.98%	17.94%

General population (women)	18.20%	6.27%	14.97%	42.36%	33.52%

General population Age 16-44	4.19%	1.21%	4.05%	22.36%	19.70%

General population Age 45-65	17.63%	4.13%	14.13%	42.99%	32.90%

General population Age ≥ 65	40.08%	16.80%	31.58%	58.72%	35.99%

General population Seniors I Age 66-74	35.27%	10.04%	24.77%	56.18%	34.31%

General population Seniors II Age 75-84	45.51%	22.60%	36.84%	63.75%	38.36%

General population Seniors III Age ≥ 85	61.54%	48.72%	66.67%	60.53%	38.36%

Source: own elaboration from Canary Island Health Survey

**Table 3:** Canarian Population that suffer a specific disease.

Population	Sample	Average	Standard deviation	Percentile 25%	Percentile 50%	Percentile 75%
**HIV+^i^**	**538**	**0.810400**	**0.2464732**	**0.749**	**0.8771**	**1**

HIV-asymptomatic	255	0.8270694	0.2362902	0.7814	0.9095	1

HIV-symptomatic	136	0.8375243	0.2075094	0.749	0.9095	1

AIDS	147	0.7563361	0.2870125	0.6533	0.8644	1

**Alzheimer disease^ii^**	**237**	**0.0958835**	**0.3872881**	**-0.153**	**0.0279**	**0.3388**

AD mild	49	0.524851	0.2501451	0.2558	0.6022	0.7485

AD medium	91	0.1817604	0.3133704	-0.068	0.1095	0.3388

AD severe	97	-0.2013763	0.2349101	-0.395	-0.241	-0.017

**Stroke^iii^**	**423**	**0.4718158**	**0.4388945**	**0.0658**	**0.5698**	**0.8265**

Stroke survivor first year	89	0.4960685	0.4245884	0.1485	0.6149	0.8265

Stroke survivor second year	193	0.4696021	0.4407007	0.0607	0.5698	0.8644

Stroke survivor three or more years	141	0.4596021	0.4474767	0.1095	0.5698	0.8265

**Diabetes Mellitus^iv^**	**358**	**0.6934785**	**0.3270208**	**0.5192**	**0.8265**	**1**

**Rheumatism; arthritis; degenerative osteoarthritis^iv^**	**1009**	**0.6874559**	**0.312844**	**0.5192**	**0.7996**	**0.8771**

**Back pain^iv^**	**997**	**0.7334693**	**0.3111258**	**0.5388**	**0.8771**	**1**

**Heart problems^iv^**	**336**	**0.6876655**	**0.3224886**	**0.5192**	**0.78415**	**0.9095**

**Osteoporosis^iv^**	**198**	**0.6325652**	**0.3275672**	**0.4186**	**0.7308**	**0.8771**

**Anxiety/depression^4v^**	**717**	**0.6630417**	**0.323674**	**0.4558**	**0.8265**	**0.9095**

**Respiratory Tract Diseases^iv^**	**210**	**0.7075705**	**0.3278174**	**0.5192**	**0.8265**	**1**

**Digestive diseases^iv^**	**481**	**0.7360424**	**0.3092**	**0.5698**	**0.8644**	**1**

EQ-5D-Spanish TTO index score or tariff.

Sources: ^i ^Observational study on HIV/AIDS (19); ^ii ^Observational study on Alzheimer's disease (18); ^iii ^Observational study on Stroke (20); ^iv ^Canary Island Health Survey

**Table 4:** Canary Island General population- EQ-5D-Spanish TTO Tariff

Population	Sample	Average	Standard deviation	Percentile 25%	Percentile 50%	Percentile 75%
General population	4282	0.8509447	0.2497144	0.8265	1	1

General population (men)	1783	0.8882825	0.224256	0.8771	1	1

General population (women)	2499	0.8243046	0.2632207	0.7869	0.9095	1

General population Age 16-44	2140	0.9226352	0.1678222	0.9095	1	1

General population Age 45-64	1156	0.824119	0.2572352	0.7869	0.9095	1

General population Age ≥ 65	986	0.7267993	0.3237708	0.5967	0.8265	1

General population Seniors I Age 65-74	598	0.7700843	0.2865273	0.7039	0.8771	1

General population Seniors II Age 75-84	316	0.6822646	0.3520769	0.5192	0.8265	1

General population Seniors III Age ≥ 85	72	0.5627514	0.4026511	0.33045	0.6528	0.87985

Source: own elaboration from Canary Island Health Survey

**Table 5:** Canarian people that suffer a specific disease.

Population	Sample	Average	Standard deviation	Percentile 25%	Percentile 50%	Percentile 75%
**HIV+^i^**	**519**	**71.14258**	**21.83456**	**60**	**75**	**90**

HIV-asymptomatic	249	74.46185	21.20806	60	80	90

HIV-symptomatic	133	66.68421	24.70902	55	70	85

AIDS	137	69.43796	18.98795	55	70	80

**Alzheimer disease^ii^**	**237**	**40.98312**	**19.47618**	**30**	**40**	**50**

AD mild	49	52.20408	18.61449	40	50	60

AD medium	91	42.8022	17.08165	30	45	50

AD severe	97	33.60825	19.06161	20	35	50

**Stroke^iii^**	**423**	**53.68618**	**26.28795**	**35**	**50**	**75**

Stroke survivor first year	89	55.95556	26.62301	40	60	70

Stroke survivor second year	193	51.64433	27.04482	30	50	70

Stroke survivor three or more years	141	55.02797	24.98082	40	50	80

**Diabetes Mellitus^iv^**	**342**	**47.81871**	**27.34437**	**30**	**50**	**70**

**Rheumatism; arthritis; degenerative osteoarthritis^iv^**	**953**	**47.28122**	**28.06338**	**20**	**50**	**70**

**Back pain^iv^**	**956**	**50.91109**	**29.65606**	**25**	**55**	**75**

**Heart problems^iv^**	**308**	**47.30519**	**27.43576**	**20**	**50**	**65**

**Osteoporosis^iv^**	**196**	**45.61735**	**27.10741**	**20**	**50**	**70**

**Anxiety/depression^4v^**	**676**	**48.43195**	**28.91151**	**20**	**50**	**70**

**Respiratory Tract Diseases^iv^**	**202**	**49.98515**	**28.27661**	**30**	**50**	**70**

**Digestive diseases^iv^**	**474**	**50.32068**	**30.08477**	**10**	**58**	**75**

Visual Analogical Scale (thermometer).

Sources: ^i ^Observational study on HIV/AIDS (19); ^ii ^Observational study on Alzheimer's disease (18); ^iii ^Observational study on Stroke (20); ^iv ^Canary Island Health Survey

**Table 6:** Canary Island general population

Population	Sample	Average	Standard deviation	Percentile 25%	Percentile 50%	Percentile 75%
General population	4176	59.3125	30.99565	40	70	80

General population (men)	1739	64.64347	29.17882	50	75	85

General population (women)	2437	55.50841	31.69165	30	60	80

General population Age 16-44	2128	65.04229	31.28681	50	80	90

General population Age 45-65	1130	56.4177	30.0071	40	65	80

General population Age ≥ 65	918	49.59368	28.5296	25	50	70

General population Seniors I Age 65-74	568	50.27641	28.75174	30	55	70

General population Seniors II Age 75-84	300	49.15	28.46545	20	55	70

General population Seniors III Age ≥ 85	50	44.5	26.25172	20	50	63

Visual Analogical Scale (thermometer)

Source: own elaboration from Canary Island Health Survey

Depression/anxiety is the most affected dimension of HRQOL for HIV and anxiety/depression patients, representing a relative younger patient group. In the Alzheimer's disease (AD), the high percentage of moderate-severe problems stands out in each one of the five dimensions (5D), especially in usual activities and in self care, 95% and 85% respectively. Stroke patients also present high percentages of severe-moderate problem in all dimensions, over the 50% in most cases. Osteomuscular diseases (rheumatism; arthritis; degenerative osteoarthritis; osteoporosis and back pain) show a similar prevalence of severe-moderate problems in the five dimensions, being pain/discomfort the most problematic dimension in people that suffer these diseases. Regarding other studied diseases such as diabetes, heart problems, anxiety/depression, respiratory and digestive diseases, severe-moderate problems are mainly present in pain-discomfort and depression/anxiety dimensions.

Focusing on disease progression, HIV and AD show a similar pattern: the higher the disease severity the higher the complications rate. However, the condition of patients who survive a stroke does not improve with time. On the contrary, the health status seems to get worse (see table 1).

As expected, comparing the results of general population (see table 2) with the results obtained for people that suffer each specific disease, it can be observed a higher percentage of people reporting moderate or severe problems in different Health Related Quality of Life dimensions for all diseases than in general population (up to 10 percentage points in 4 of the 5 studied dimensions). Alzheimer's disease and Stroke patients suffer the highest loss in HRQOL, with differences exceeding 30 percentage points in 3 of the 5 dimensions. AD is the most remarkable case of HRQOL loss due to the existing differences between patients and general population, approximately 60 percentage points in self-care and usual activities. The percentage of people that survive a stroke reporting moderate or severe problems in different HRQOL dimensions is remarkable. Likewise, rheumatism and diabetes show differences in problems reported in the five dimensions of 10 percentage points. Digestive and heart problems present this type of differences in problems reported in 3 of the 5 dimensions. People that suffer other diseases reported lower differences in moderate or severe problems in HRQOL dimensions compared with the general population.

The most discouraging results in percentile analysis correspond to AD. Table 3 shows that the best group of AD patients, percentile 75, has a low TTO value, 0.3388, and this index score or tariff takes a negative value in percentiles 25. Stroke results show that some patients in the percentile 75 almost recover the normal QOL after stroke; however, a considerable number of patients (percentiles 50 and 25), suffered severe consequences after the cerebrovascular accident. HIV/AIDS results are fairly better compared with other diseases. In the other diseases, we observe a progressive loss of QOL compared to general population, that is, that percentile 75 shows a similar behaviour, whereas percentile 25 has values that are slightly lower. On the one hand, percentile 75 of HIV, diabetes, back pain, respiratory and digestive disease seems to have a similar QOL compared to general population. On the other hand, rheumatism, heart problems, osteoporosis and anxiety have after-effects and show differences of about a 10 percentage points in percentile 75 (see tables 3 and 4).

Results obtained by VAS method for specific diseases and general population are similar to TTO ones (see tables 5 and 6). AD has again the lowest values in VAS results but these numbers are higher than those obtained by TTO method for AD. This situation recurs in the stroke case. In the case of anxiety/depression, back pain and rheumatism, the loss of QOL is progressive; that is, there is small differences between QOL of general population and people with specific diseases who reported better health status (up to 5-10 points approximately in percentile 75) and this difference increases in people who reported worse health status (up to 15-20 points in percentile 25). Osteoporosis shows also a progressive pattern with a slight difference, a group of patients, percentile 75, maintain the QOL of general population. Percentile 75 and 50 of digestive disease show that an important number of patients that almost maintain a normal life, whereas patients in percentile 25 suffer severe consequences. Diabetes, heart problems and respiratory diseases patients have a loss of QOL that is constant across the percentile analysis, or slightly increases in percentile 25.

## Discussion and conclusions

Over the years, there has been a progressive interest in listening user's and citizens' voice in different aspects of the delivery of health services. The identification and assessment of HRQOL of patients and the general population are a promising way of achieving this goal. Health surveys offer the opportunity to monitor population's health problems by means of validated instruments and to assess its potential impact on HRQOL. From a public health perspective, such monitoring allows the identification of potential changes in prevalence and inequalities on health status, and reveals unmet needs in the community [27].

The impact of health state changes on an individual's quality of life has gained increased attention in social and medical clinical research [28]. There is an extended acknowledgement that "classic" measurement of health as expectancy of life and morbidity rates should be complemented, especially in developed countries with a high and increasing prevalence of chronic conditions, by Health Related Quality of Life measurements.

The Canary Island Health Survey gives an overview of the Canary citizens' health status to joining two types of indicators: self perceived health status (HRQOL) and chronic conditions (self-reported, but based on known medical diagnosis). However, this useful information should be complemented with "ad hoc" studies focused on diseases with strong health and social impact but low rates of prevalence.

In this work, we show that depression/anxiety and pain/discomfort are the most affected dimensions in the Canary population that suffer a chronic disease. The progression pattern observed is the higher severity of disease higher probability of reporting moderate or severe problems in different HRQOL dimensions, with the exception of stroke patients that don't seem to improve with the passage of time.

The HRQOL monitoring in the general population requires generic instruments that ideally capture all-important aspects of self perceived health, allowing comparisons within and between populations. The combination of EQ-5 D with any other specific scales should be carefully considered. Specific measurements bring into focus the burden on health and functioning for a health condition or treatment. Generic HRQOL measurements are intended to provide information on general function and well-being with the advantage of allowing comparisons among different diseases or populations. Besides EQ-5 D can be used to estimate and compare self-perceived effectiveness and cost-effectiveness of different health care interventions intended to improve populations' health [29-31]. Hence, the EQ-5 D is one of the instruments most frequently used in cost utility analysis for the development of QALYs in the field of Health Technology Assessment [31]. Although some countries have expressed criticism of the use of QALYs in economic evaluation [32,33], the outcome remains the most demanded by the rating agencies of health interventions in most European countries [34-39].

In this sense, the measurement of populations' HRQOL from a country or region and the study of its evolution can be a useful tool for decision-makers. Self perceived health status can contribute to complement the information reported by life expectancy and incidence and prevalence morbidity indicators. A complete description on the health status of citizens can help to an efficient allocation of health care and social resources in order to satisfy the social needs. Besides, having a synthetic indicator that combine expectancy and quality of life make easier the comparison between costs and consequences of implementing health policies. For instance, policies to prevent infant obesity, restrictive laws on tobacco and alcohol consumption, the implementation of integrated programmes on Ischemic Heart Diseases, Tumours, Stroke, Mental Illness, Diabetes Mellitus, or the expansion of another preventive programs, only for mentioning some of the most recent health policies promoted by the Spanish Ministry of Health and Social Policy jointly with regional authorities. So, the measurement of self-perceived health of the population using multidimensional concepts should be considered as a relevant part of the development of methods and tools that could help to a better understanding of the effectiveness of health care services and to a more appropriated valuation of the returns of the health care systems.

Certain limitations of this study should be discussed. First, like most other studies on general population, our analysis does not include institutionalized people. Second, it can be argued that data on HRQOL are self-reported and that fact limits its validity. However, HRQOL is the way of getting information on subjective aspects of health. So, as Sullivan (2003) [1] note "...patient outcomes... are valid because they are subjective". Third, illnesses were self-reported in CIHS. Although, other studies show evidence of good agreement between self-reporting and clinical diagnoses of chronic diseases [40-42], the replies of people that had been diagnosed can be affected by the accessibility or availability of medical services when they were asked about their diseases, . In second place, we have performed a descriptive study instead of developing a statistical model that helping to explain differences in HRQOL between individuals. Unfortunately, we do not have a collection of same explanatory variables in the observational studies and CIHS. Only age and sex/gender and diagnosed diseases could have been used in this analysis. For this reason, at the moment, we considered more interesting to show, in a descriptive way, the HRQOL of people that suffer a chronic disease in comparison with general population. Other studies analyzed the association between HRQOL and socioeconomic health determinants in Canary Island [43] using more sophisticated statistical techniques [43]. Finally, the different data sources evaluated in the paper were developed at different time frames. Positive, or negative, changes in health habits trends and the introduction of new health care technologies can improve, or worse, the health status of population and the self perceived health status of people that suffer a certain disease. However, in our study the differences between the dates where studies and Canary Island Health Survey were developed are small, from 2001 to 2004, and we would not expect sharp change in self perceived health status of people that suffer a certain disease.

Despite these limitations, this study shows a remarkable loss of HRQOL in people that suffer a chronic disease compared to general population. These findings stress the importance of disease prevention interventions as well as the early detection (screening) and efficient management of chronic conditions, in order to improve HRQOL. Future research is needed for improving our knowledge about explanatory variables that affect the HRQOL of people along their lifetime.

## Abbreviations

5D: Five dimensions; AD: Alzheimer's disease; AIDS: Acquired immune deficiency syndrome; CDC: Center of Disease Control and Prevention; CDR: Clinical Dementia Rating; CIHS: Canary Island Health Survey; HIV: Human Immunodeficiency Virus; HRQOL: Health-related Quality of Life; QOL: Quality of Life; TTO: Time Trade-Off; VAS: Visual Analogue Scale.

## Competing interests

The authors declare that they have no competing interest.

## Authors' contributions

JOM and JLB contributed to the design of the study, analysis of the results and writing of the manuscript. MWC contributed to the analysis of the results and writing of the manuscript. PSA contributed to the design of the study and writing of the manuscript. All authors read and approved the final manuscript.

## Pre-publication history

The pre-publication history for this paper can be accessed here:

http://www.biomedcentral.com/1471-2458/10/675/prepub

## References

[B1] Sullivan M The new subjective medicine: taking the patient's point of view on health care and health Social Science & Medicine 2003 56 1595 1604 10.1016/s0277-9536(02)00159-4 12614708

[B2] Wood-Dauphine S Assessing quality of life in clinical research: From where have we come and where are we going? Journal of Clinical Epidemiology 1999 52 355 363 10.1016/S0895-4356(98)00179-6 10235176

[B3] World Health Organization The constitution of the World Health Organization 1947 1 WHO Chronicle 6 24

[B4] Testa MA Simonson DC Assessment of Quality-of-Life Outcomes N Eng J Med 1996 334 835 840 10.1056/NEJM199603283341306 8596551

[B5] Testa MA Interpretation of quality-of-life outcomes Medical Care 2000 38 II166 II174 10.1097/00005650-200009002-00026 10982103

[B6] Idler EL Benyamini Y Self-rated health and mortality: A review of twenty-seven community studies Journal of Health and Social Behaviour 1997 38 21 37 10.2307/2955359 9097506

[B7] Ries A Kaplan RM Limbreg TM Prewitt LM Effects of pulmonary rehabilitation on physiologic and psychosocial outcomes in patients with chronic obstructive pulmonary disease Annals of Internal Medicine 1995 122 823 832 7741366 10.7326/0003-4819-122-11-199506010-00003

[B8] Rodriguez-Artalejo F Guallar-Castillon P Pascual CR Otero CM Montes AO Garcia AN Conthe P Chiva MO Banegas JR Herrera MC Health-related quality of life as a predictor of hospital readmission and death among patients with heart failure Arch Intern Med 2005 165 11 1274 9 10.1001/archinte.165.11.1274 15956007

[B9] Kaplan MS Berthelot JM Feeny D McFarland BH Khan S Orpana H The predictive validity of health-related quality of life measures: mortality in a longitudinal population-based study Qual Life Res 2007 16 9 1539 1546 10.1007/s11136-007-9256-7 17899447

[B10] Conelli JE Philbrick JT Smith GR Kaiser DL Wymer A Health perceptions of primary care patients and the influence on health care utilization Medical Care 1989 27 S99 S109 10.1097/00005650-198903001-00009 2921890

[B11] Siu AL Reuben DB Ouslander JB Osterweil D Using multidimensional health measures in older persons to identify the risk of hospitalization and skilled nursing placement Quality of Life Research 1993 2 253 261 10.1007/BF00434797 8220360

[B12] Saarni SI Sintonen H Suvisaari J Koskinen S Aromaa A Lönnqvist J The impact of 29 chronic conditions on health-related quality of life: a general population survey in Finland using 15 D and EQ-5D Qual Life Res 2006 15 8 1403 14 10.1007/s11136-006-0020-1 16960751

[B13] Hisashige A Mikasa H Katayama T Description and valuation of health-related quality of life among the general public in Japan by the EuroQoL The Journal of Medical Investigation 1998 45 123 9 9864973

[B14] Johnson JA Coons SJ Ergo A Szava-Kovats G Valuation of EuroQoL (EQ-5D) health states in an adult US sample Pharmacoeconomics 1998 13 421 33 10.2165/00019053-199813040-00005 10178666

[B15] Kind P Dolan P Gudex C Williams A Variations in population health status: results from a United Kingdom national questionnaire survey British Medical Journal 1998 316 736 41 9529408 10.1136/bmj.316.7133.736 PMC28477

[B16] Rabin R de Charro F EQ-5D: A measure of health status from the EuroQol Group Annals of Medicine 2001 33 5 337 343 10.3109/07853890109002087 11491192

[B17] Shaw JW Johnson JA Coons SJ US valuation of the EQ-5 D health states: Development and testing of the D1 valuation model Medical Care 2005 43 3 203 220 10.1097/00005650-200503000-00003 15725977

[B18] López-Bastida J Perestelo L Serrano P Oliva-Moreno J Socioeconomic costs and quality of life of people with Alzheimer disease in Canary Island Neurology 2006 67 2186 2191 10.1212/01.wnl.0000249311.80411.93 17190942

[B19] López-Bastida J Oliva J Perestelo L Serrano P Direct and Indirect Costs in Ambulatory patients living with HIV/AIDS BMC Health Serv Res 2009 9 55 10.1186/1472-6963-9-55 19331682 PMC2670289

[B20] Lopez-Bastida J Serrano Aguilar P Monton Alvarez F The economic burden of stroke in Spain Value Health 2003 6 615

[B21] Brooks R EuroQol: the current state of play Health Policy 1996 37 53 72 10.1016/0168-8510(96)00822-6 10158943

[B22] Badia X Schiaffino A Alonso J Herdman M Using the EuroQol 5 D in the Catalan general population: feasibility and construct validity Qual Life Res 1998 7 311 322 10.1023/A:1008894502042 9610215

[B23] Badia X Roset M Herdman M Kind P A comparison of United Kingdom and Spanish general population time trade-off values for EQ-5 D health states Medical Decision Making 2001 21 1 7 16 10.1177/0272989X0102100102 11206949

[B24] Dolan P Sutton M Mapping visual analogue scale health state valuations onto standard gamble and time trade-off values Social Science & Medicine 1997 44 19 1519-1530 10.1016/s0277-9536(96)00271-7 9160441

[B25] Bleichrodt H Johannesson M Standard gamble, time trade-off and rating scale: experimental results on the ranking properties of QALYs Journal of Health Economics 1997 16 155 175 10.1016/S0167-6296(96)00509-7 10169092

[B26] Arnesen T Trommald M Are QALYs based on time trade-off comparable? A systematic review of TTO methodologies Health Economics 2005 14 39 53 10.1002/hec.895 15386674

[B27] Nordlund A Ekberg K Kristenson M EQ-5 D in a general population survey--a description of the most commonly reported EQ-5 D health states using the SF-36 Qual Life Res 2005 14 4 1099 109 10.1007/s11136-004-3062-2 16041905

[B28] Schwartz CE Sprangers MAG Methodological approaches for assessing response shift in longitudinal health-related quality-of-life research Social Science & Medicine 1999 48 1531 1548 10.1016/s0277-9536(99)00047-7 10400255

[B29] Kind P Dolan P Gudex C Williams A Variations in population health status:results from a United Kingdom national questionnaire survey BMJ 1998 316 7133 736 41 9529408 10.1136/bmj.316.7133.736 PMC28477

[B30] Gold MR Siegel JE Russell LB Weinstein MC (eds) Cost-Effectiveness in Health and Medicine. Report of the Panel on Cost-Effectiveness in Health and Medicine 1996 New York: Oxford University Press

[B31] National Institute for Clinical Excellence (NICE) Guide to the Methods of Technology Appraisals 2008

[B32] Porzsolt F Ackermann M Amelung V The value of health care--a matter of discussion in Germany BMC Health Serv Res 2007 7 1 10.1186/1472-6963-7-1 17199886 PMC1764870

[B33] Schwarzer R Siebert U Methods, procedures, and contextual characteristics of health technology assessment and health policy decision making: comparison of health technology assessment agencies in Germany, United Kingdom, France, and Sweden Int J Technol Assess Health Care 2009 25 3 305 14 10.1017/S0266462309990092 19619349

[B34] Siebert M Clauss LC Carlisle M Casteels B de Jong P Kreuzer M Health Technology assessment for medical devices in Europe. What must be considered Int J Technol Assess Health Care 2002 18 733 740 12391964

[B35] Claxton K Sculpher M Drummond M A rational framework for decision making by the National Institute for Clinical Excellence (NICE) Lancet 2002 360 711 715 10.1016/S0140-6736(02)09832-X 12241891

[B36] Davies L Drummond M Papanikolaou P Prioritizing investments in health technology assessment. Can we assess potential value for money? Int J Technol Assess Health Care 2000 16 73 91 10.1017/S0266462300016172 10815355

[B37] Detsky AS Laupacis A Relevance of cost-effectiveness analysis to clinicians and policy makers JAMA 2007 298 2 221 4 10.1001/jama.298.2.221 17622605

[B38] Rawlins M Culyer AJ National Institute for Clinical Excellence and its value judgments BMJ 2004 329 224 7 10.1136/bmj.329.7459.224 15271836 PMC487742

[B39] Drummond M Jones AM Economic evaluation and decision-makers The Elgar companion to health economics 2006 Cheltenham: Edward Elgar 537 45

[B40] Orfila F Ferrer M Lamarca R Tebe C Domingo-Salvany A Alonso J Gender differences in health-related quality of life among the elderly: the role of objective functional capacity and chronic conditions Soc Sci Med 2006 63 9 2367 80 10.1016/j.socscimed.2006.06.017 16884840

[B41] Bush TL Miller SR Golden AL Hale WE Self-Report and medical record report agreement of selected medical conditions in the elderly self-report and medical record report agreement of selected medical conditions in the elderly American Journal of Public Health 1989 79 11 1554 1556 10.2105/AJPH.79.11.1554 2817172 PMC1349815

[B42] Bombard JM Powell KE Martin LM Helmick CG Wilson WH Validity and reliability of self-reported arthritis: Georgia senior centers, 2000-2001 American Journal of Preventive Medicine 2005 28 3 251 258 10.1016/j.amepre.2004.12.004 15766612

[B43] Oliva-Moreno J Zozaya N Lopez-Varcarcel BG Opposite poles: A comparison between two Spanish regions in health related quality of life, with implications for health policy BMC Public Health 2010 10 576 10.1186/1471-2458-10-675 20868523 PMC2955693

